# American Dental Association

**Published:** 1884-11

**Authors:** Chas. Mayr


### AMERICAN DENTAL ASSOCIATION.

### TWENTY-FOURTH ANNUAL MEETING, AT SARATOGA.

REPORTED FOR THE INDEPENDENT PRACTITIONER BY PROF. CHAS. MAYR.

(Continued from page 564.)

### WEDNESDAY, AUGUST 16TH.

### MORNING SESSION.

The meeting was called to order at 10 o'clock A. M.; President Darby in the chair. The privileges of the floor were voted to Prof.

Chas. Mayr, of Springfield, Mass., and to Drs. Bazin, Lovejoy, Borden and Andres, of Montreal.

The discussion of the paper by Dr. Harlan, upon Pyorrhœa Alveolaris, was declared in order.

*Dr. Shepard*—Hoped that speakers would be exact in their expressions. He himself had said yesterday, "dilute sulphuric acid." That was inexact. He should have given the strength. He intended to have added, "in the proportion of one part of sulphuric acid to three of water."

*Dr. Atkinson*—If the speaker meant one part of acid to three of water, that would be much stronger than the tincture of *aromatic* sulphuric acid.

*Dr. Shepard*—I meant aromatic sulphuric acid.

*Dr. Atkinson*—Then the gentleman was not exact when demanding that others should be.

*Dr. Searle*—I love to ask the questions why, and what. In the first place, why do we use these various remedies? I think we have not gone deep enough into the nature of the disease. We hear it said sometimes that decalcification and caries are synonymous; also Pyorrhœa and decalcification. They are both diseases of the soft tissues. The lime salts have nothing to do with them. If I have rightly studied Pyorrhœa, I should declare it a disease of the pericementum. Originally it is set in action by some irritant, and that irritant may be tartar, low organisms, or mechanical irritation; but whatever may be the cause, the disease starts in the pericementum, and works itself far down between the alveolus and the pericementum before it makes itself manifest at all. The disease has been operating, usually, for a long time before we notice it, and unless we can apply our remedies to the special part affected by the disease, we might just as well throw them on the sidewalk. We all know that ptyalism and the various affections of the mucous membrane are not Pyorrhœa. We know that inflammation is not the disease in question until it progresses to a certain stage. Now, where does Pyorrhœa commence? We cannot define exactly at what point of the inflammation of the periodonteal membrane the disease commences, but it is probably during the stage when the pericementum itself becomes involved in the disease. To cure the disease we have to remove all foreign matter; we must go down to the point of union between healthy and diseased tissue, and treat it as a disease; not as an injury. How shall we treat it? After removal of foreign matter we dress it like any other wound, as nearly antiseptically as possible; whether it is a germ disease or not I do not know, but in some cases it may be, and deserves as much an antiseptic treatment as any similar disease. The best antiseptic dressing I am acquainted with is carbolic acid, full strength, applied, as stated last night, far down to the point of union between the diseased and healthy tissues. What more can you do? The teeth cannot rest; you cannot apply a plaster, as to an ulcer of the skin; in ulcers around teeth the carbolic acid itself will form a coating by coagulation, which will keep out extraneous matter. How about repeating the treatment every few days? I would say let it alone, most severely. Having once performed an operation, amputated a limb and dressed it, let it get well alone; give nature a chance; do not disturb it. Somebody spoke of the application of external friction; this, I think, is perhaps the best way of removing food, foreign substances, etc., that lodge accidentally between the alveolus and the roots of the teeth. Let your patient place the finger low down, and move it up so as to compress the tissues, and then rinse the mouth with a bucketful of water.

*Dr. Stockton*—It does not seem necessary to say any more about the subject, as any one who kept his ears open last evening evidently knows everything about it. One gentleman, very prominent in the profession, said it was caused by deposits, and could be cured by remedies; another said it was not caused by deposits, and could not be cured by remedies; one said a saturated solution of an acid would cure it; the other said there was no such thing as a saturated solution. I do not know any more about it than I did before coming. I thank Dr. Harlan for his paper, and hope he will give us the etiology of the disease another year.

*Dr. Hunt*—I consider the disease constitutional, and think people and tribes who live simply in accordance with the laws of nature, have better teeth than the inhabitants of so-called civilized countries. I also consider the disease one of the symptoms of urinary troubles.

*Dr. Searle*—Cases at which I had been pegging away for five or eight years, have yielded in a short time to strong carbolic acid.

*Dr. Pierce*—I did not mean to say that urinary calculus was the cause of Pyorrhœa, but the systemic condition which is cause for one disease, is also for the other. There are cases of pus in the alveolar socket, due to local causes; these removed, the disease will subside. Pyorrhœa is hereditary in many cases; I think if inquiries are made among patients, you will hear them tell that "father and mother lost their teeth in the same way;" "all the teeth of father and mother came out without decay, all sound," etc. I do not believe in the transmission of disease, but of tendencies.

*Dr. Watkins*—Dr. Pierce thinks the disease cannot be cured; I would like to relate a case where, I think, I cured it. It was one of the lateral incisors, elongated, pressed out of position, surrounded with pus pockets everywhere, except in one small space on the labial surface; the tooth was very loose; I examined it and found a very little tartar about half way up the root; I removed this and the border of the alveolus, which was necrosed; I applied aromatic sulphuric acid, full strength. A week afterwards the patient came again, and I found that I had overlooked a small spot; I treated this in the same manner, and dismissed the patient; this was three years ago, and the tooth is now sound in its socket.

*Dr. How*—Exhibited a few instruments; one a syringe with a fine platinum tube, the other a scaler to remove deposits by pushing or pulling, and the sides shaped so as to scrape the diseased alveolus.

*Dr. Harlan*—There is a wide difference between salivary calculus and Pyorrhœa. In the first affection the pericementum has not been detached beyond the line of deposit. In any event the salivary deposit is all around the neck of the teeth, and continuous, not broken up into islands. The pericementum cannot be lifted from the root of the tooth beyond the line of salivary deposits. Whatever may be the initiative of Pyorrhœa, if mechanical, chemical, traumatic, or anything else, it has a beginning at the gingival margin, on the ligament surrounding the neck of the tooth. It is believed, in connection with this injury, that micro-organisms of a peculiar form are found; that they manufacture a soluble ferment, which destroys the tissues in advance. If that theory be correct, it is necessary that a remedy to destroy these micro-organisms should be injected. The reason for the frequent injection is, that the remedy you use may destroy the developing organisms, but not destroy the spores, so that in three or four days it would be necessary to repeat the injection, and so on until all spores have been destroyed; Dr. Black, of Jacksonville, has advocated this theory. It is no wonder that Dr. Pierce comes to the conclusion that the disease is not curable, if he uses only one remedy which, as he himself says, cannot be applied in all cases. I have received hundreds of letters from all over the United States; I have visited the colleges, and their officers have told me that they were trying the treatment I recommended. They have asked me to see the patients, and when I asked for the remedies and instruments, to show how to manipulate the one and to use the others, they did not have them. How can a gentleman expect to accomplish anything unless he himself will faithfully try and be honest? It is no criticism against a method or treatment if the criticiser himself does not practice or attempt to practice what is advocated.

*Dr. Brown*—There seems to be two grounds taken in this discussion; the one that Pyorrhœa can be cured, the other that it cannot. Now, let us take an intermediate ground. Who would think to condemn a physician, because, when he cured a sore throat, it recurs in a year, nor, if any one had cured Pyorrhœa Alveolaris, and the tendency or disease reappeared in three or four years. It may take generations of treatment to cure disease and eradicate it. The child says, "mother's teeth dropped out, and sister's did; I come to you fearing the disease." The question is not whether it can be cured permanently. Dr. Harlan is right; he has cured cases; so have I, and so have we all; but can one tell what the cases will be five years hence? Many will recur. Does a man allow his wooden house to burn down because it caught fire three or four times, while his neighbor's stone house never burned?

*Dr. Shepard*—From my observation I think the disease can be wiped out in the first generation. Taken in the incipient stages it is as curable as any disease; there will be no loss of the alveolar process. Patients should bring their children to us in early life; the tendencies, as Dr. Pierce said, are hereditary. We need to watch them. The term Pyorrhœa, meaning originally pus-flow, is used to mean the loss of tissue and the ensuing inflammation. The section of nomenclature has not been active in the proper direction.

*Dr. Atkinson*—We have heard a great deal of good sense and of nonsense; some have classified diseases in the plural before they have defined them in the singular, and they have got so tangled in perception that we have had a hodge-podge of brilliant perceptions so intermingled as to be made worthless. Let us get our minds to the point of what a disease is, and how it acts, and whether it is capable of being limited or annihilated. There is such a thing as antidoting of diseased activity, which is the result of the introduction of foreign elements into the functioning body. Every phlegmonous abscess is self-limiting and self-curing. It will go in the direction of the least resistance. If the flow of pabulum by pressure is prevented, it will abort. Why does not Pyorrhœa Alveolaris heal? It is the introduction of micro-organisms which float in the air that set up a ferment adverse to the formation of tissues, so that they can be built after the type of the tissue. I cured a case of twenty-four years' standing in eight days. I then used wood creosote. What is recurrence? It is either repetition of imbecility on the part of the investigator, or malevolence by persisting in a wrong position. These cases of which we speak as recurrences are simply territories, only enclosed a little, but not healed, in which the bacteria had sufficient oxygen to keep them alive, and they broke down the tissue, until it made its way by the recuperative law and broke through on to the surface; or it took another way of curing, which is encystment, whereby the foreign body becomes a mineral in its function, like a bullet in the body, remaining encysted for years. I have in my own person a splinter of wood that has become resorbed so much that I can find it only occasionally. When you have removed the body inimical to the organization, that organization alone does the cure. Anything may be according to the organization a food, a poison, or a remedy. On motion the subject was passed.

(TO BE CONTINUED.)

